# RNF90 negatively regulates cellular antiviral responses by targeting MITA for degradation

**DOI:** 10.1371/journal.ppat.1008387

**Published:** 2020-03-03

**Authors:** Bo Yang, Yue Liu, Yuhan Cui, Di Song, Ge Zhang, Shujun Ma, Yanzi Liu, Mengmeng Chen, Fan Chen, Hui Wang, Jie Wang

**Affiliations:** 1 Henan Key Laboratory of immunology and targeted drug, Xinxiang Medical University, Xinxiang, Henan Province, China; 2 Henan Collaborative Innovation Center of Molecular Diagnosis and Laboratory Medicine, School of Laboratory Medicine, Xinxiang Medical University, Xinxiang, Henan Province, China; 3 Department of Laboratory Medicine, the Third Affiliated Hospital of Xinxiang Medical University, Xinxiang, Henan Province, China; Florida State University, UNITED STATES

## Abstract

Mediator of IRF3 activation (MITA, also named as STING/ERIS/MPYS/TMEM173), is essential to DNA virus- or cytosolic DNA-triggered innate immune responses. In this study, we demonstrated the negative regulatory role of RING-finger protein (RNF) 90 in innate immune responses targeting MITA. RNF90 promoted K48-linked ubiquitination of MITA and its proteasome-dependent degradation. Overexpression of RNF90 inhibited HSV-1- or cytosolic DNA-induced immune responses whereas RNF90 knockdown had the opposite effects. Moreover, RNF90-deficient bone marrow-derived dendritic cells (BMDCs), bone marrow-derived macrophages (BMMs) and mouse embryonic fibroblasts (MEFs) exhibited increased DNA virus- or cytosolic DNA-triggered signaling and RNF90 deficiency protected mice from DNA virus infection. Taken together, our findings suggested a novel function of RNF90 in innate immunity.

## Author summary

After the invasion of viruses, the genetic materials, RNA and DNA generated by the viruses, could be recognized by the cytosolic RNA or DNA sensors and then these sensors will initiate the innate immune signaling pathway and protect the host from viral invasion. However, excessive immune responses may cause tissue damage and do harm to the host. Therefore, the innate immune responses should be tightly controlled and turned off timely. MITA is a key adaptor in DNA-sensor signaling pathway. Here, we find that RNF90, a known E3 ubiquitin-protein ligase, interacted with MITA, enhanced the ubiquitination of MITA and promoted the degradation of MITA. RNF90-defient mice showed increased innate immune responses upon DNA virus HSV-1 infection. Our research revealed a new negative regulatory mechanism of the host to manipulate the antiviral innate immune responses.

## Introduction

The host innate immune system serves as the first line to eliminate viral invasions. The initiation of antiviral innate immune responses relies on the recognition of pathogen-associated molecular patterns (PAMPs) from invading viruses by a series of pattern recognition receptors (PRRs) [1]. Viral nucleic acids, including genomic DNA or RNA, transcripts, and replicative intermediates, are very important PAMPs and could be sensed by cytosolic RNA or DNA sensors [2]. In most cell types, RIG-I-like receptors (RLRs), including retinoic-acid-inducible protein I (RIG-I) and Melanoma differentiation-associated gene 5 (Mda5), serves as intracellular viral RNA receptors [3], whereas cytosolic DNA forms of viruses could be recognized by an array of molecules identified as cytosolic DNA sensors [4], such as DNA-dependent activator of IFN regulatory factors [5], RNA polymerase III [6], IFN-γ-inducible factor 16 [7], DExD/H-box helicase 41 [8], Ku70 [9], cyclic GMP–AMP (cGAMP) synthase (cGAS) [10], and so on. These RNA or DNA receptors recognized the invasion of viruses and triggered the antiviral signaling pathways to produce type I IFN and proinflammatory cytokines, leading to the activation of downstream antiviral innate immune responses to limit the replication of viruses and construct the antiviral microenvironment [11].

Mediator of IRF3 activation (MITA), also called stimulator of interferon genes (STING), endoplasmic reticulum interferon stimulator (ERIS), MPYS (a novel plasma membrane tetraspanner) and Transmembrane protein 173 (TMEM173), plays a very important role in the antiviral signaling pathways, which serves as an adaptor protein for both RNA viruses- and DNA viruses-induced antiviral immune responses [12–15]. Upon RNA virus infection, MITA interacts with RIG-I and its downstream adaptor protein, virus-induced signaling adaptor (VISA), and recruits TANK-binding kinase 1 (TBK1) to the VISA-associated complex to activate interferon regulatory factor (IRF)3 [12]. Depletion of MITA exhibited impaired immune response against RNA viruses in a virus- and cell type-specific manner. MITA-deficient mice were defective in antiviral signaling pathways to some RNA viruses [16]. Meanwhile, the effect of MITA in cytosolic DNA sensors-mediated signaling pathways is more universal [17]. A lot of cytosolic DNA sensors, such as cGAS, IFI16 and Ku70, signal through MITA [18, 19]. Based on our current knowledge, cGAS is the best characterized cytosolic DNA sensor in multiple cell types. Upon binding to dsDNA, cGAS catalyzes the formation of the second messenger cyclic GMP-AMP (cGAMP), which will activate MITA to generate type I interferon (IFN) and subsequent innate immune responses [20]. MITA-deficient mice showed impaired production of type I IFN in response to DNA virus infection and cytosolic DNA stimulation, thus susceptible to lethal infection with DNA viruses, including HSV-1, vaccinia virus, and murine gammaherpesvirus 68 (MHV68) [16].

Excessive innate immune responses will do harm to the host and may cause tissue damage. Therefore, the host immune system is tightly controlled by various regulatory strategies to adjust the strength and duration of the immune responses [21]. Protein posttranslational modifications (PTMs) form part of this exquisite regulatory system and modulate the immune function in a timely and efficient way, including phosphorylation, ubiquitination, glycosylation, S-nitrosylation, methylation, acetylation and lipidation [22]. Among these modifications, ubiquitination is well characterized and has been proved to play a critical role in the antiviral signaling pathway by modulating the activation, stability, affinity and location of the targeting key proteins in the pathways [23]. Ubiquitin, the 76-amino acid polypeptide universally expressed in most tissues of eukaryotic organisms, has seven Lys residues (K6, K11, K27, K29, K33, K48, and K63), each of which could link the ubiquitin to the targeting proteins [24]. K48-and K63-linked ubiquitination are the most studied types of linkage. K48-linked ubiquitination usually mediates the degradation of protein in a proteasome-dependent way, whereas K63-linked modification mainly regulates signal transduction in a non-proteolytic manner [25].

The modification of ubiquitination involves a cascade of chemical reactions catalyzed sequentially by E1 ubiquitin-activating enzyme, E2 ubiquitin-conjugating enzymes and E3 ubiquitin-protein ligases. Till now, in human genome, only 2 E1 ubiquitin-activating enzyme and 38 E2 ubiquitin-conjugating enzymes have been reported. However, more than 600 E3 ubiquitin-protein ligases have been identified, which determines the high substrate specificity for the protein ubiquitination [26]. E3 ubiquitin-protein ligases contain three main classes, HECT-, RING- and RBP-type. The activity of RING-type ubiquitin-protein ligases relies on their RING-domains which contain conserved cysteine and histidine residues essential for recruiting the E2 ubiquitin-conjugating enzymes [27]. Several RING-type E3 ubiquitin-protein ligases have been demonstrated in the regulation of antiviral responses [28]. For example, RNF5 regulates antiviral responses by targeting MITA for degradation after viral infection [29], whereas TRIM32 targets MITA for K63-linked ubiquitination to positively modulate type I IFN production [30]. RNF90, also named as TRIM7/GNIP, is a known RING-type E3 ubiquitin-protein ligases, which mediates K63-linked ubiquitination of the AP-1 coactivator RACO-1 [31]. Recently, RNF90 was reported to facilitate the TLR4-mediated innate response [32].

Here, our findings demonstrated a negative regulatory role of RNF90 in DNA virus- or cytosolic DNA-triggered innate immune responses, which targeted MITA and promoted its K48-linked ubiquitination, leading to the degradation of MITA. RNF90-deficient bone marrow-derived dendritic cells (BMDCs), bone marrow-derived macrophages (BMMs) and mouse embryonic fibroblasts (MEFs) exhibited enhanced DNA virus- or cytosolic DNA-triggered signaling and RNF90 deficiency protected mice from DNA virus infection. Thus, our study identified a new strategy for host cells to accurately regulate innate immune response after viral infection.

## Results

### RNF90 negatively regulates exogenous cytosolic DNA-induced innate immune responses

**Fig 1:**
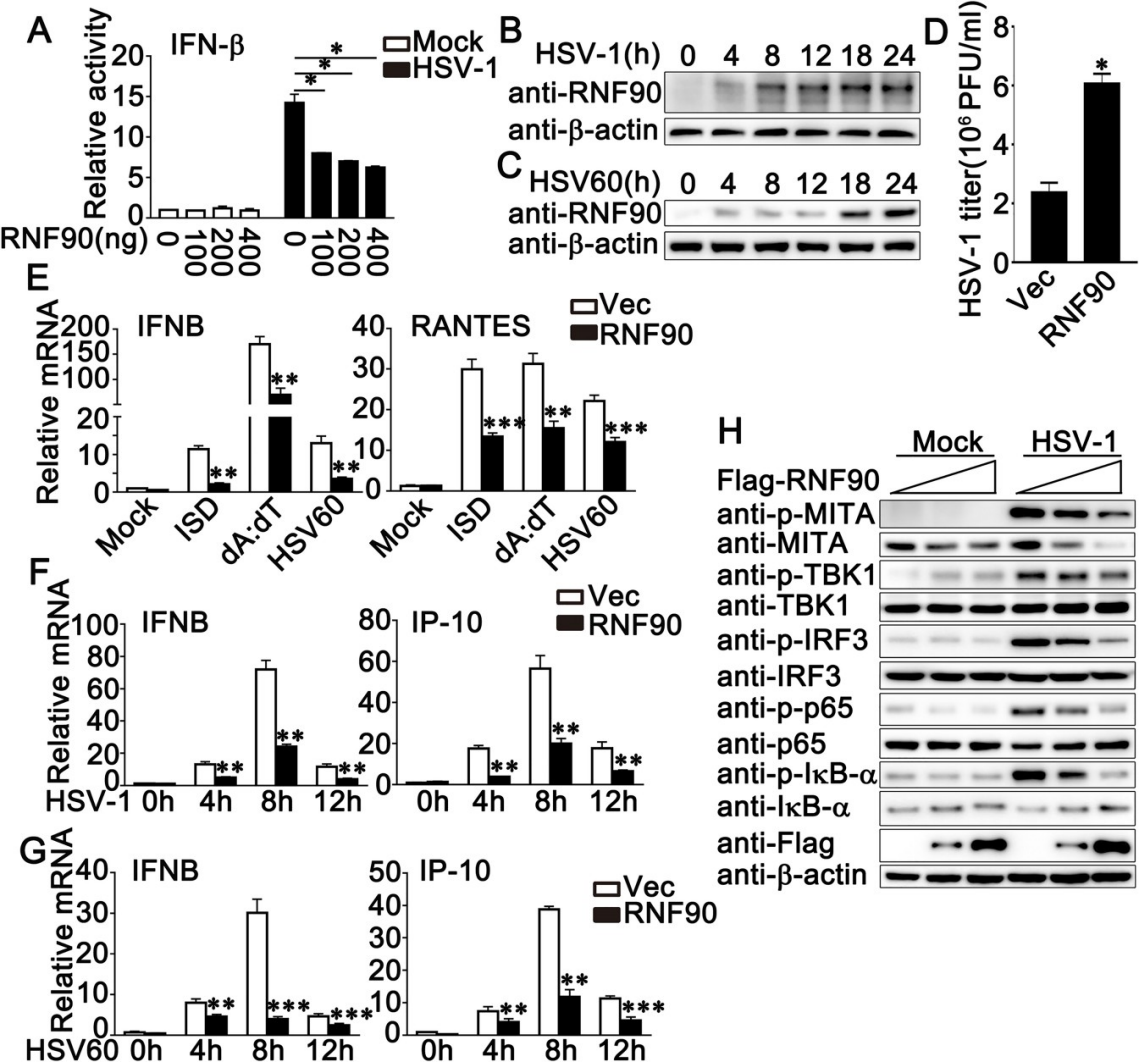
RNF90 overexpression inhibits exogenous cytosolic DNA- induced innate immune responses. (A) Luciferase activity in HEK293 cells transfected with an IFN-β luciferase reporter, together with the increasing amounts of RNF90 plasmid as indicated, and then infected with HSV-1 or left untreated (Mock) for 24 h. (B, C) PMA-THP1 cells were stimulated with HSV-1(B) or 1 μg/ml HSV60 (C) for indicated periods of time. Afterwards, the cells were lysed for immunoblot assays. β-actin was used as a loading control in all the immunoblot assays. (D) HaCaT keratinocytes were transfected with the empty vector (Vec) or the RNF90 plasmid and then infected with HSV-1 for 24 h. The titers of HSV-1 were determined by standard plaque assay. (E) HaCaT keratinocytes were transfected with the empty vector (Vec) or the RNA90 plasmid, and then stimulated with ISD (1 μg/ml), poly(dA:dT) (1 μg/ml), and HSV60 (1 μg/ml) for 8 h. Then the cells were lysed for real-time PCR analyses. (F, G) HaCaT keratinocytes cells were transfected with the empty vector (Vec) or the RNA90 plasmid, and then stimulated with HSV-1 (F) or HSV60 (1 μg/ml) (G) for indicated periods of time. Then the cells were lysed for real-time PCR analyses. (H) HaCaT keratinocytes cells were transfected with the empty vector (Vec) or the RNA90 plasmid (0, 0.4, 0.8 μg), and then infected with HSV-1 for 8 h. Afterwards, the cells were lysed for immunoblot assays. β-actin served as a loading control in all the immunoblot assays. The data are representative of three independent experiments and are presented as mean ± SEM. *p < 0.05, **p < 0.01, ***p < 0.001.

To determine whether the RNF protein family played a role in DNA virus-triggered immune responses, we assessed the effects of the RNF protein family members on the IFN-β promoter reporter activity upon HSV-1 infection. The luciferase assay results revealed that RNF90 overexpression in HEK293 cells inhibited HSV-1-induced IFN-β activity in a dose-dependent manner (Fig 1A). We confirmed the result in HaCaT keratinocytes, which were the target cells and first point of contact for a variety of DNA viruses. As shown in S1A Fig, RNF90 overexpression in HaCaT cells inhibited HSV-1-induced IFN-β, ISRE or NF-κB reporter activity in a dose-dependent manner. Next, we examined the relationship between RNF90 expression pattern and stimulation by cytosolic DNA or DNA virus. Immunoblot results indicated that in Phorbol-12-myristate-13-acetate (PMA)-differentiated THP1 (PMA-THP1, a human macrophage-like cell line) cells, both HSV-1 infection and viral DNA HSV60 transfection could induce RNF90 expression (Fig 1B and 1C). To confirm the role of RNF90 in HSV-1 infection, we investigated whether RNF90 was able to regulate HSV-1 infection. RNF90 was overexpressed in HaCaT cells, and the supernatants were subjected to the standard plaque assay. As shown in Fig 1D, the RNF90 overexpression increased the titers of HSV-1, suggesting RNF90 negatively regulated the antiviral immune responses upon HSV-1 infection. Next, we examined whether RNF90 had a similar role upon the stimulation of cytosolic DNA. Real-time PCR results of IFN-β and RANTES indicated that the innate immune responses against cytosolic DNA stimulation, including ISD, poly (dA:dT) and HSV-60, were inhibited by RNF90 overexpression (Fig 1E). RNF90 overexpression also inhibited cGAMP-induced IFN-β and RANTES production (S1B Fig). To confirm these results, we checked different periods of time after stimulation, and the inhibitory effect of RNF90 overexpression on HSV-1 or HSV60-induced innate immune responses was observed at 4, 8, 12 h after stimulation (Fig 1F and 1G). In addition, RNF90 overexpression decreased the phosphorylation of MITA, TBK1, IRF3 and p65, which were triggered by HSV-1 infection (Fig 1H). Taken together, our results suggested a negative regulatory role of RNF90 in exogenous cytosolic DNA-induced innate immune responses.

**Fig 2:**
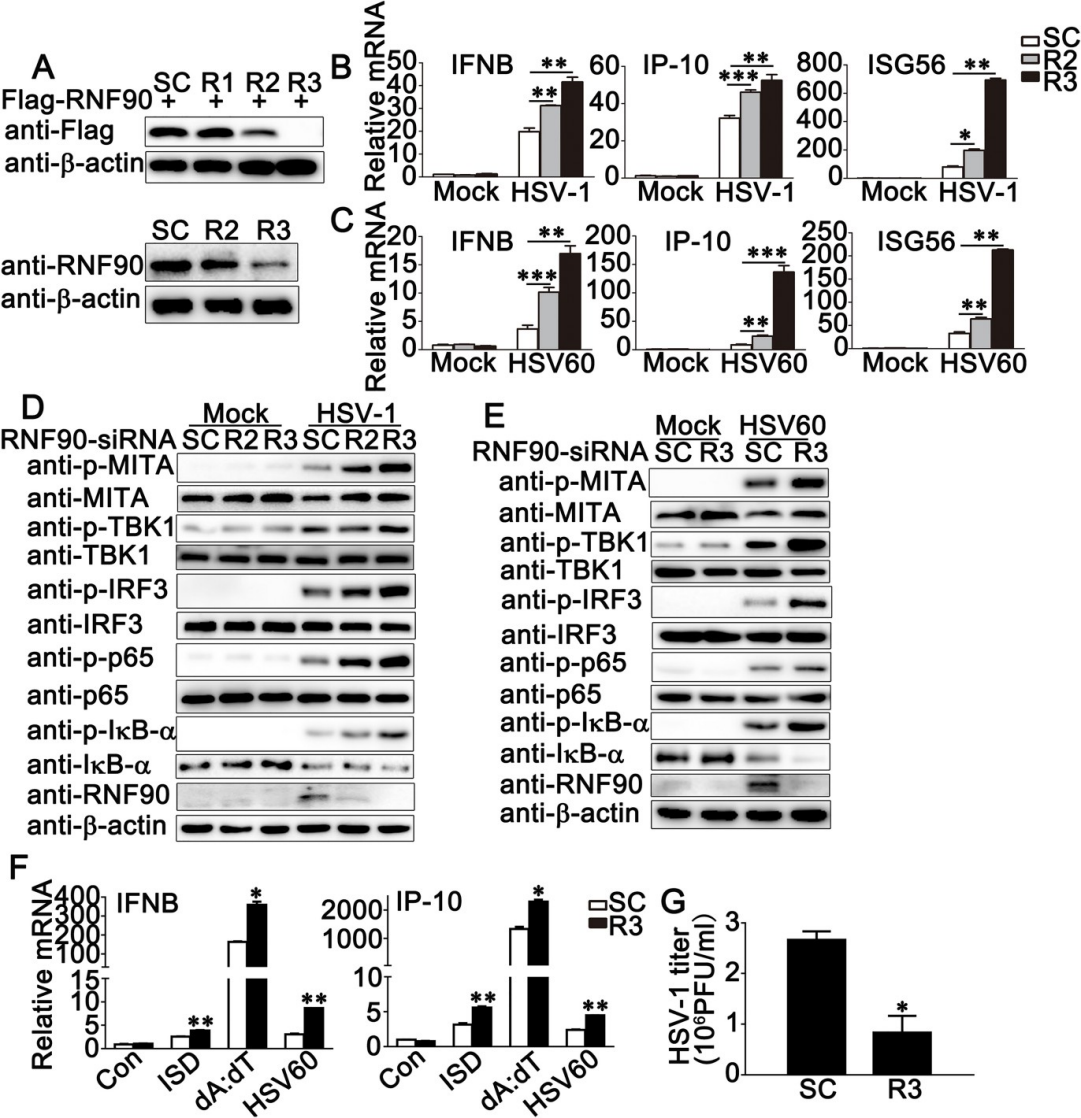
RNF90 knockdown promotes exogenous cytosolic DNA- induced innate immune responses. (A) HEK293 cells were transfected with Flag-RNF90, and then transfected with control siRNA (SC) or RNF90-specific siRNA (R1, R2 and R3). At 24 h after transfection, the cells were lysed for immunoblot assays (top). PMA-THP1 cells were transfected with control siRNA (SC) or RNF90-specific siRNA (R2 and R3). At 24 h after transfection, the cells were infected with HSV-1 for 8 h, and then immunoblot assay was performed (bottom). (B, C) PMA-THP1 cells were transfected with control siRNA (SC) or RNF90-specific siRNA (R2 and R3) for 24 h, and then stimulated with HSV-1 (B) or HSV60 (C) for 8 h. The cells were lysed for real-time PCR analysis. (D) PMA-THP1 cells were transfected with control siRNA (SC) or RNF90-specific siRNA (R2 and R3) for 24 h, and then infected with HSV-1 for 4 h. The cells were lysed for immunoblot assays. (E) PMA-THP1 cells were transfected with control siRNA (SC) or RNF90-specific siRNA (R3) for 24 h, and then transfected with HSV-60 for 4 h. The cells were lysed for immunoblot assays. (F) PMA-THP1 cells were transfected with control siRNA (SC) or RNF90-specific siRNA (R3) for 24 h, and then transfected with ISD (1μg/ml), poly(dA:dT) (1μg/ml), and HSV60 (1μg/ml) for 8 h. Then the cells were lysed for real-time PCR analyses. (G) PMA-THP1 cells were transfected with control siRNA (SC) or RNF90-specific siRNA (R3) for 24 h, and then infected with HSV-1 for 24 h. The titers of HSV-1 were determined by standard plaque assay. β-actin served as a loading control in all the immunoblot assays. The data are representative of three independent experiments and are presented as mean ± SEM. *p < 0.05, **p < 0.01, ***p < 0.001.

Next, we used the knockdown approach to further identify the role of endogenous RNF90 in DNA virus- or cytosolic DNA-triggered innate immune responses. We designed three pairs of siRNA oligonucleotides specific for RNF90 RNA (R1, R2, and R3) and a control siRNA (SC). As shown in Fig 2A, R2 and R3 inhibited both exogenous and endogenous RNF90 expression, and R3 showed higher efficiency in inhibition than R2 (Fig 2A). Therefore, both R2 and R3 were used in the following experiments. In PMA-THP1 cells, RNF90 knockdown by R2 or R3 promoted the production of IFN-β, IP-10 and ISG56 in mRNA levels upon HSV-1, HSV60 or cGAMP stimulation and the effects of R3 were more significant than R2, which was consistent with their inhibitory efficiency on RNF90 expression (Fig 2B and 2C and S1C Fig). Moreover, RNF90 knockdown increased the phosphorylation of MITA, TBK1, IRF3 and p65, which were triggered by HSV-1 infection or HSV-60 transfection (Fig 2D and 2E). Similar results were obtained from HaCaT cells (S2A and S2B Fig). Next, we examined the role of RNF90 knockdown in other types of cytosolic DNA-triggered immune responses. Real-time PCR results indicated that RNF90 knockdown increased IFN-β and IP-10 expression which were induced by cytosolic DNA ISD or poly (dA:dT) transfection (Fig 2F). Finally, plaque assay results suggested RNF90 knockdown downregulated HSV-1 infection (Fig 2G). Taken together, our results suggested a negative regulatory role of RNF90 in exogenous cytosolic DNA-induced innate immune responses.

### RNF90 interacts with MITA

**Fig 3:**
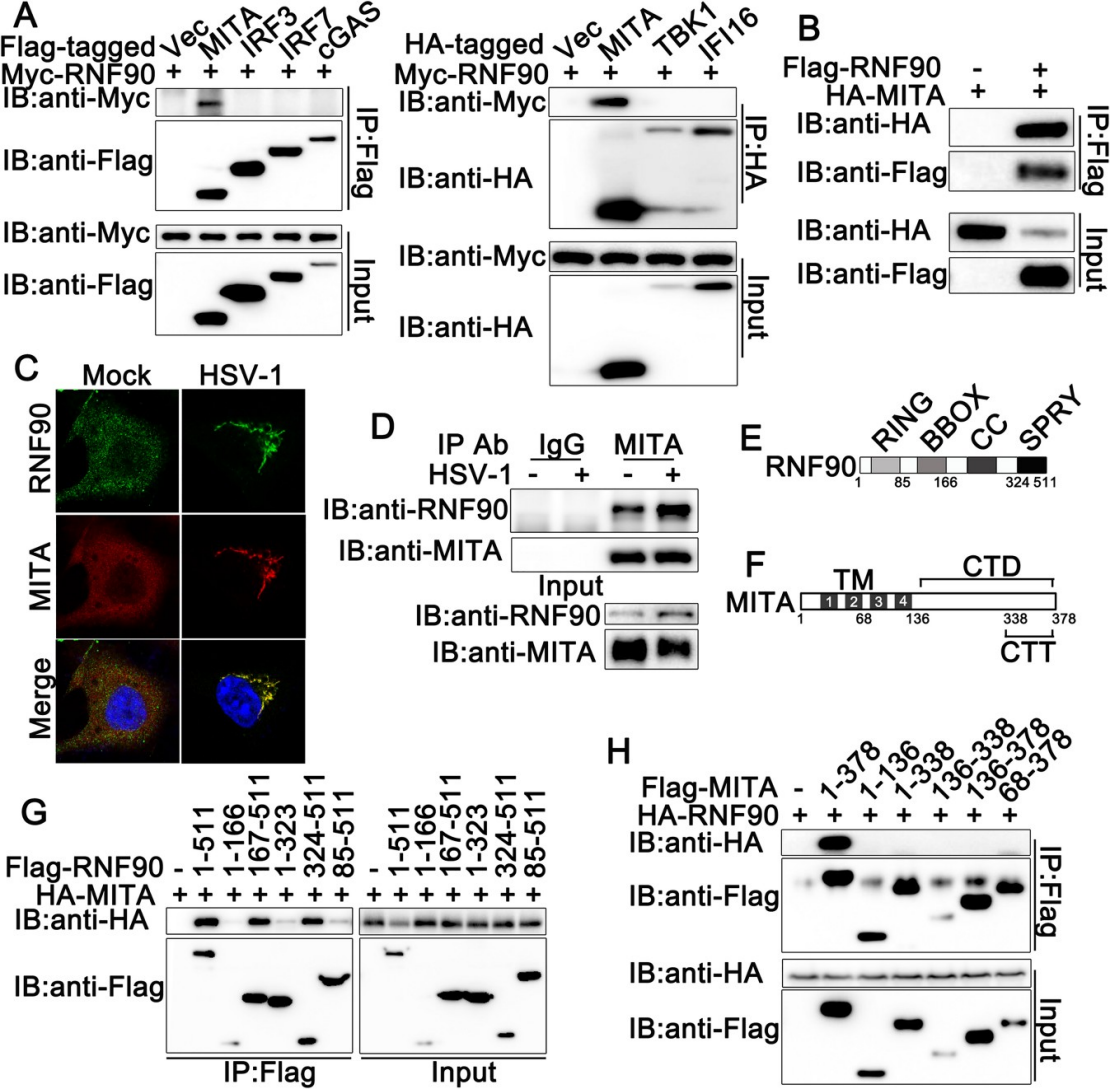
RNF90 interacts with MITA. (A) HEK293 cells were transfected with indicated plasmids. At 24 h after transfection, the cell lysates were subjected to immunoprecipitation (IP) and immunoblot (IB) analysis as indicated. (B) HEK293 cells were transfected with Flag-RNF90 and HA-MITA. At 24 h after transfection, the cell lysates were subjected to immunoprecipitation (IP) and immunoblot (IB) analysis as indicated. (C) HaCaT keratinocytes were transfected with expressing plasmids for HA-MITA and Flag-RNF90. At 24 h after transfection, HaCaT keratinocytes were stimulated with HSV-1 or left untreated for another 8 h. Immunofluorescence was performed using anti-HA (red) and anti-Flag (green). Nuclei were stained with DAPI. (D) PMA-THP1 cells were infected with HSV-1 for 8h, and then the cell lysates were subjected to immunoprecipitation (IP) and immunoblot (IB) analysis as indicated. (E) A schematic presentation of full-length RNF90 and its mutants. RING, ring-finger domain; BBOX, B-box domain; CC, coiled-coil domain; SPRY, SPRY domain. (F) A schematic presentation of full-length MITA and its mutants. TM, Transmembrane; TM1, 21-41aa; TM2, 47-67aa; TM3, 87-106aa; TM4, 115-135aa; CTD, carboxy-terminal domain; CTT, carboxy-terminal tail. (G) HEK293T cells were transfected with indicated plasmids. At 24 h after transfection, the cell lysates were subjected to immunoprecipitation (IP) and immunoblot (IB) analysis as indicated. (H) HEK293T cells were transfected with indicated plasmids. At 24 h after transfection, the cell lysates were subjected to immunoprecipitation (IP) and immunoblot (IB) analysis as indicated. The data are representative of three independent experiments.

To elucidate the molecular mechanisms responsible for the negative regulatory role of RNF90 in cytosolic DNA-induced innate immune responses, we performed immunoprecipitation assays to identify whether RNF90 interacted with the signal molecules involved in cytosolic DNA-triggered signaling pathway. We observed RNF90 could only be co-immunoprecipitated with MITA, but not with cGAS, IFI16, TBK1, IRF3 and IRF7 (Fig 3A and 3B), suggesting exogenous expressed RNF90 interacted with MITA. This interaction was also suggested by the co-localization of RNF90 with MITA using confocal experiments (Fig 3C). Moreover, the interaction between endogenous expressed RNF90 and MITA could be observed in PMA-THP1 cells with or without HSV-1 infection (Fig 3D). Finally, we tried to figure out the region of RNF90 responsible for its interaction with MITA. As shown in Fig 3E and 3G, we observed residues 324–511 still was able to interact with MITA, but not the residues 1–323, suggesting the SPRY domain was responsible for the interaction with MITA. Interestingly, residues 85–511 had only a much weaker ability to bind MITA, suggesting RNF90 might contain some special structure served as inhibitory regulation for the interaction with MITA. We also mapped the binding regions on MITA for RNF90 association. As shown in Fig 3F and 3H, only full-length MITA coimmunoprecipitated with RNF90, suggesting each part of MITA might contribute to its interaction with RNF90. Taken together, our findings suggested RNF90 interacted with MITA.

### RNF90 inhibits MITA-mediated signaling pathway and enhances the degradation of MITA

**Fig 4:**
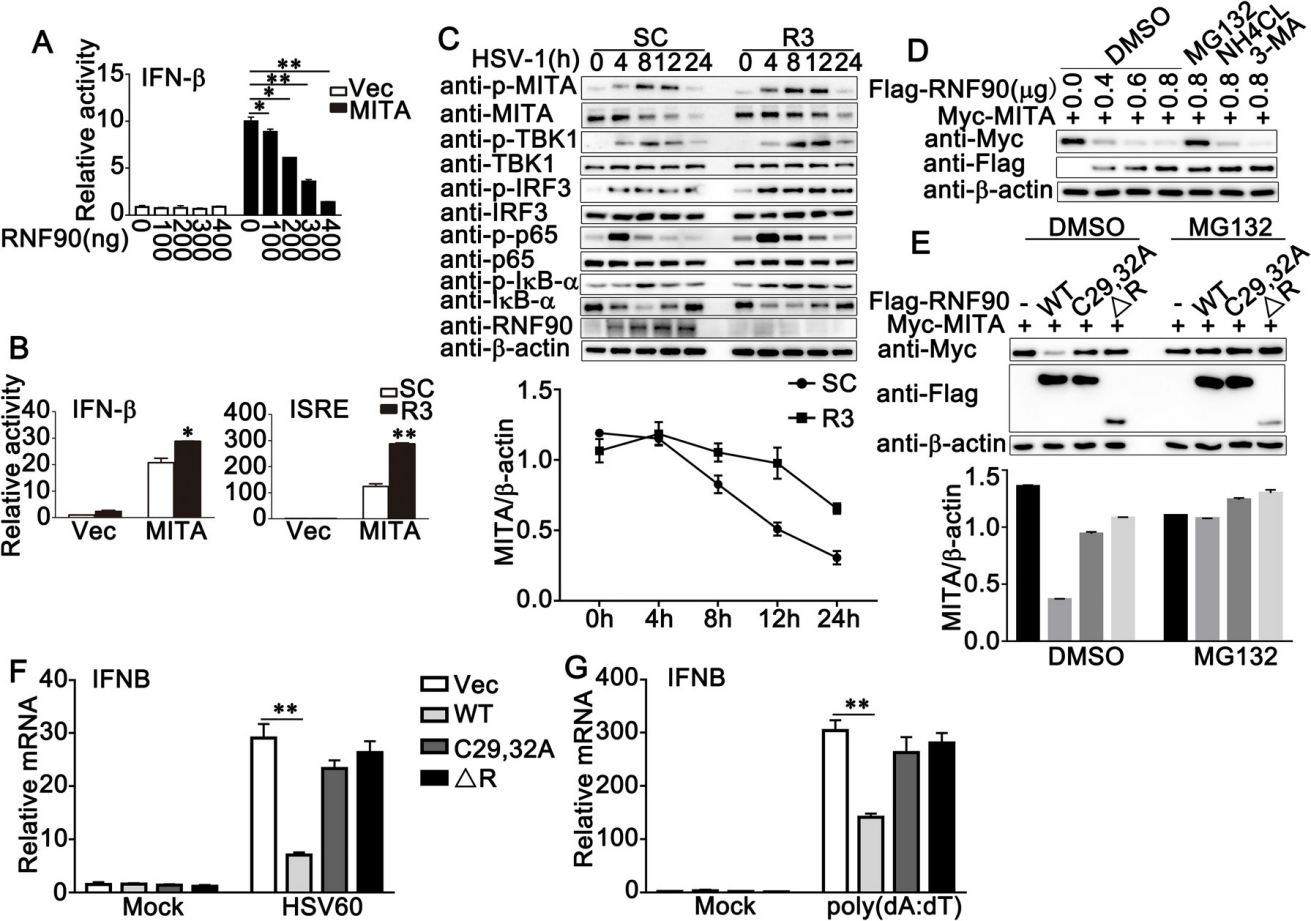
RNF90 inhibits MITA-mediated signaling pathway and enhances the degradation of MITA. (A) HEK293 cells were transfected with an IFN-β luciferase reporter, together with HA-MITA and the increasing amounts of Flag-RNF90 plasmid as indicated. At 24 h after transfection, the cells were lysed for luciferase assay. (B) HEK293 cells were transfected with control siRNA (SC) or RNF90-specific siRNA (R3), together with the MITA expressing plasmid and the IFN-β or ISRE luciferase reporter. At 24 h after transfection, the cells were lysed for luciferase assay. (C) PMA-THP1 cells were transfected with control siRNA (SC) or RNF90-specific siRNA (R3). At 24 h after transfection, the cells were stimulated with HSV-1 for indicated periods of time. The cell lysates were subjected to immunoblot assays. The quantity of MITA expression was normalized by β-actin. (D) HEK293 cells were transfected with Myc-MITA and the increasing amounts of RNF90 plasmids as indicated. 24 h later, cells were treated with DMSO, MG132 (20 mM), NH4CL (5 mM) or 3-MA (2 mM) separately for 6 h and lysed for immunoblot assays. (E) HEK293 cells were transfected with indicated plasmids for 24 h and then treated with DMSO or MG132 for 6 h. The cells were lysed for immunoblot assays. The quantity of MITA expression was normalized by β-actin. (F, G) HaCaT cells were transfected with indicated plasmids, and then stimulated with HSV60 (1 μg/ml) (F), and poly(dA:dT) (1 μg/ml) (G) for 8 h. Then the cells were lysed for real-time PCR analyses. β-actin served as a loading control in all the immunoblot assays. The data are representative of three independent experiments and are presented as mean ± SEM. *p < 0.05, **p < 0.01.

Given the fact that RNF90 interacted with MITA, we investigated whether RNF90 affected MITA-mediated signaling pathway. Luciferase assay results indicated that RNF90 overexpression inhibited MITA-induced IFN-β reporter activation in a dose-dependent manner (Fig 4A), whereas RNF90 knockdown had the opposite effects (Fig 4B). We then investigated the effects of RNF90 on signaling mediated by other signaling adaptors such as cGAS, TBK1 and IRF3. As shown in S3A Fig, overexpression of RNF90 inhibited cGAS/MITA-mediated signaling whereas no significant inhibitory effect on TBK1- or IRF3-mediated signaling was observed. Consistently, knockdown of RNF90 promoted cGAS/MITA-mediated signaling whereas no significant effect on TBK1- or IRF3-mediated signaling was observed (S3B Fig). Because RNF90 is a kind of E3 ubiquitin ligase, we examined whether RNF90 affected the stability of MITA. As shown in Fig 4C, upon HSV-1 infection, RNF90 knockdown increased MITA expression in protein levels, but not in mRNA levels (S4A Fig). Furthermore, RNF90 overexpression inhibited MITA expression in protein levels in a dose-dependent manner and this inhibition could be reversed by the proteasome inhibitor MG132, but not by NH_4_Cl or 3-MA (Fig 4D), suggesting a proteasome-dependent mechanism underlying the negative regulation of MITA expression. Because RING domain has been reported to be essential for the ubiquitin E3 ligase activity of RNF90 [31], we tested whether the C29, 32A substitution of RNF90 or the RNF90 mutant lacking RING domain (ΔR) impaired its ability to degrade MITA. We found that both C29, 32A and ΔR mutants did not efficiently inhibit MITA protein expression as did wild-type RNF90 (Fig 4E). Furthermore, as shown in Fig 4F and 4G, both C29, 32A and ΔR mutants did not efficiently inhibit MITA-mediated IFN-β production as did wild-type RNF90. Taken together, our data showed RNF90 targeted MITA for degradation to regulate the innate immune responses.

### RNF90 enhances the K48-linked ubiquitination of MITA

**Fig 5:**
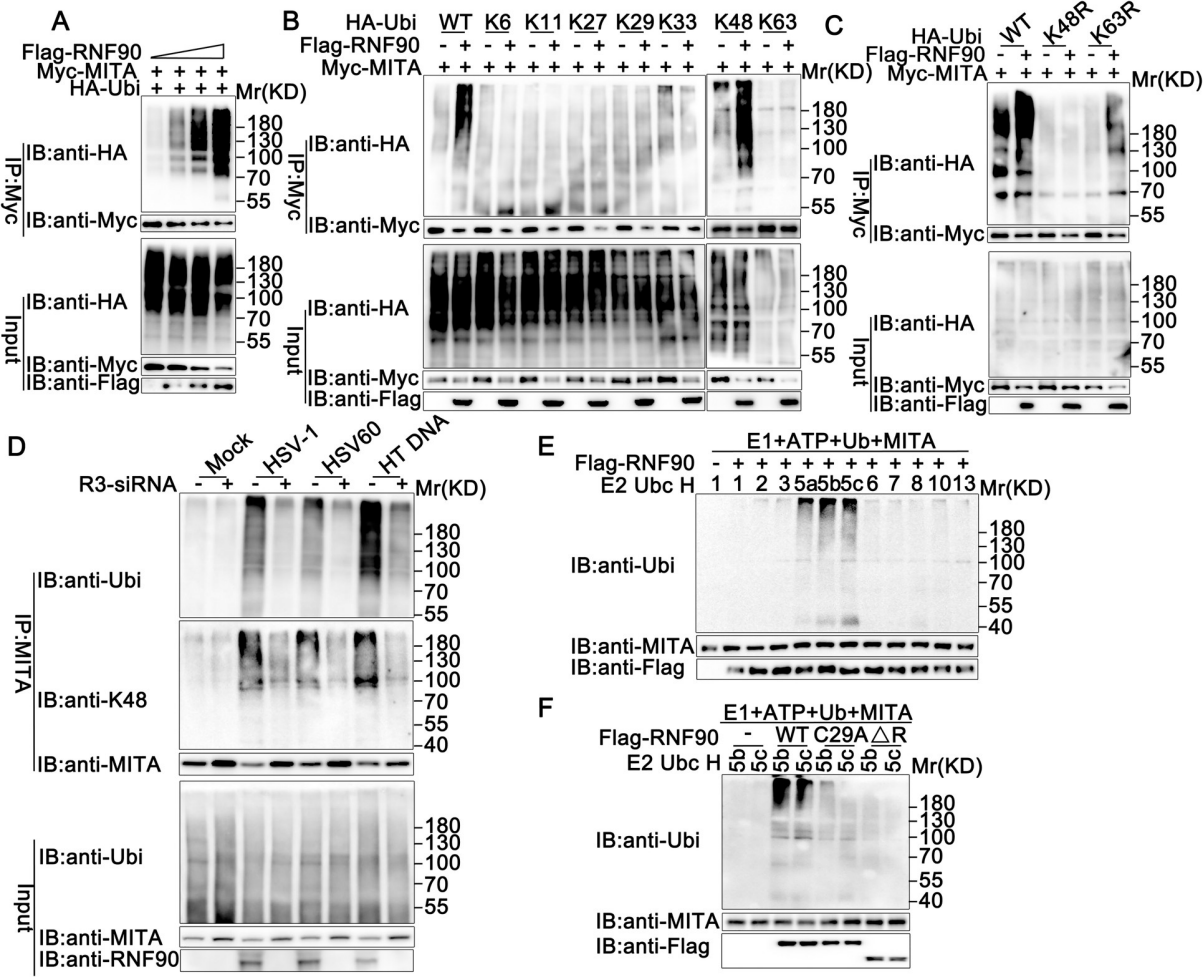
RNF90 promotes the K48-ubiquitination of MITA. (A) HEK293T cells were transfected with the indicated plasmids and increasing amounts of Flag-RNF90 (0, 0.5, 1 and 2 μg). At 24 h after transfection, the cells were lysed and subjected to immunoprecipitation (IP) and immunoblot (IB) analysis. (B) HEK293T cells were transfected with indicated plasmids. At 24 h after transfection, immunoprecipitation (IP) and immunoblot (IB) analysis were performed as indicated. (C) HEK293T cells were transfected with various combinations of plasmids as indicated. 24 h later, immunoprecipitation (IP) and immunoblot (IB) analysis were performed. (D) PMA-THP1 cells were transfected with control siRNA (SC) or RNF90-specific siRNA (R3) for 24 h, and then stimulated with HSV-1, 1 μg/ml HSV60, or 1 μg/ml HT DNA for 8 h. The cells were lysed for immunoprecipitation (IP) and immunoblot (IB) assays. (E, F) Immunoblot analysis of MITA ubiquitination in vitro. MITA and wild-type RNF90 (E) or its mutants (F) were quickly translated in vitro, and then, the biotin-ubiquitin E1 and indicated E2s were added for the in vitro ubiquitination assays. Ubiquitination of MITA was detected by anti-Ubi. The data are representative of three independent experiments.

Next, we examined whether RNF90 regulated the ubiquitination of MITA. RNF90 was co-transfected with MITA and HA-tagged ubiquitin, and the ubiquitination of MITA was detected by immunoprecipitation. As show in Fig 5A, RNF90 overexpression increased the ubiquitination of MITA in a dose-dependent manner. However, neither residues 1–166 nor residues 167–511 increased MITA ubiquitination, suggesting both the N-terminal and the C-terminal of RNF90 were important to its effect on the ubiquitination of MITA (S4B Fig). Moreover, the C29, 32A mutant could not enhance the ubiquitination of MITA (S4C Fig), indicating the RING domain of RNF90 played a big role in its function.

Ubiquitin itself has seven Lys residues (K6, K11, K27, K29, K33, K48, and K63), each of which can participate in further ubiquitination and generate seven different polyubiquitin chains [24]. To identify the type of linkage that was promoted by RNF90, we used expression plasmids for ubiquitin mutants retaining only a single lysine residue. Immunoprecipitation and immunoblot analysis indicated that RNF90 promoted only K48 mutants (only the Lys residue 48 was retained) mediated ubiquitination of MITA, suggesting RNF90 enhanced the K48-linked ubiquitination of MITA (Fig 5B). This phenomenon was further confirmed by the usage of K48R (only the Lys residue 48 was mutated to Arg) and K63R (only the Lys residue 63 was mutated to Arg). Immunoprecipitation and immunoblot analysis indicated that RNF90 increased K63R mediated ubiquitination of MITA, but not K48R, indicating the Lys residue 48 was essential to the RNF90-triggered linkage of MITA with ubiquitin (Fig 5C). Moreover, RNF90 knockdown significantly decreased the K48-mediated endogenous MITA polyubiquitination upon HSV-1, HSV60 or Herring testis (HT) DNA stimulation(Fig 5D). Next, we explored the site of STING responsible for RNF90-mediated ubiquitination. As shown in S4D Fig, the ubiquitination of mutation of K150 to arginine was obviously attenuated, indicating the K150 of MITA was responsible for RNF90-mediated ubiquitination. In addition, MITA mutant K150R-induced ISRE activity could not be affected by RNF90 (S4E Fig), suggesting the ubiquitination modification was essential to the function of RNF90 on MITA-mediated signaling pathway. Finally, in vitro ubiquitination assays suggested that RNF90 promoted the ubiquitination of MITA directly (Fig 5E and 5F). Taken together, these data suggested that RNF90 enhanced the K48-linked ubiquitination of MITA, which could be recognized and degraded by the proteasome system.

### RNF90 deficiency promotes exogenous cytosolic DNA-triggered innate immune responses in BMDCs

**Fig 6:**
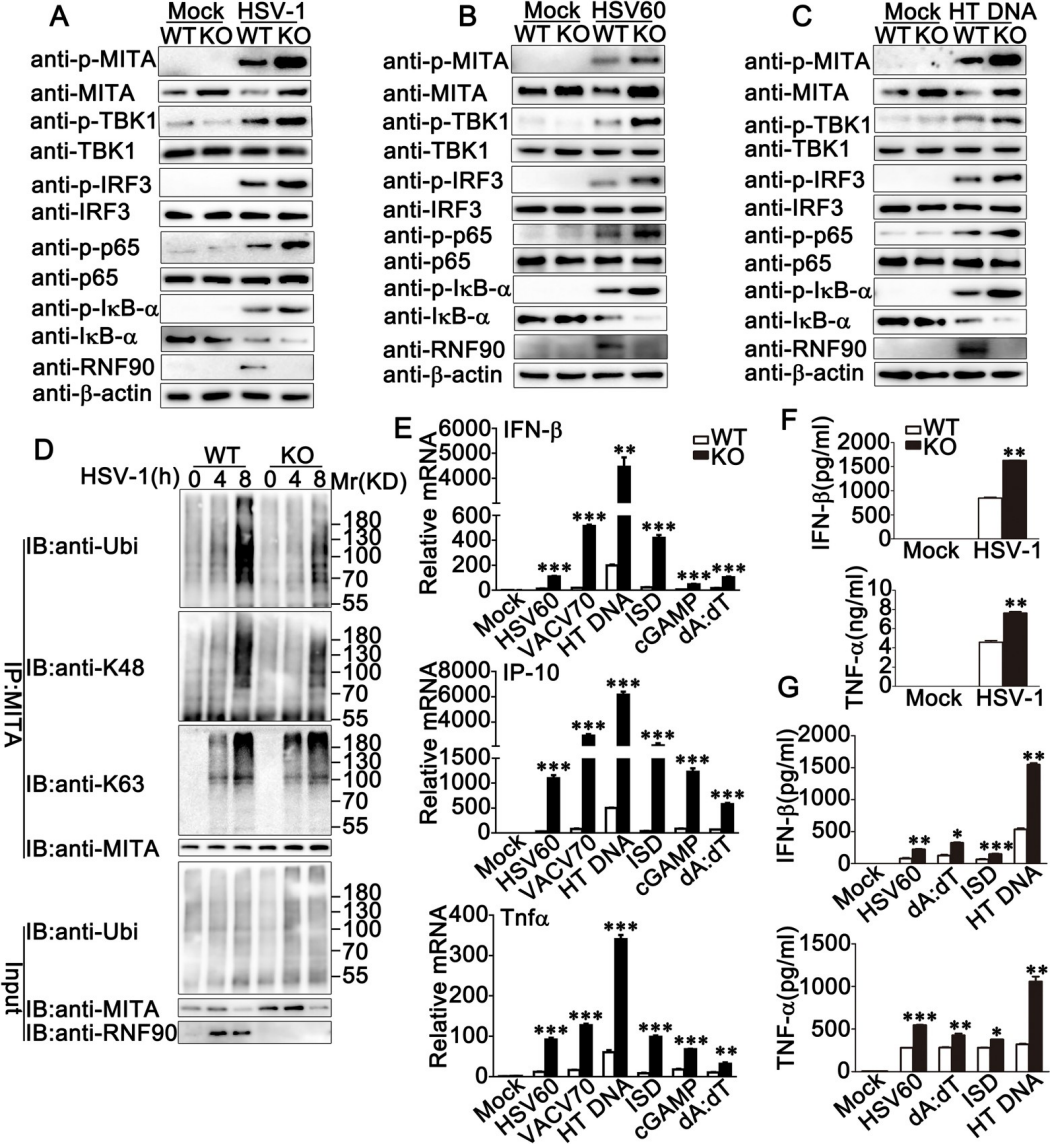
RNF90 deficiency promotes exogenous cytosolic DNA-triggered innate immune responses in BMDCs. (A) Wild-type (WT) and RNF90-deficient (KO) BMDCs were infected with HSV-1 for 8 h. The cells were lysed for immunoblot analysis. (B, C) Wild-type (WT) and RNF90-deficient (KO) BMDCs were transfected with 1 μg/ml HSV60 (B) or 1 μg/ml HT DNA (C) for 8 h. The cells were lysed for immunoblot analysis. (D) Wild-type (WT) and RNF90-deficient (KO) BMDCs were infected with HSV-1 for indicated periods of time. The cells were lysed for immunoprecipitation (IP) and immunoblot (IB) assays. (E) Wild-type (WT) and RNF90-deficient (KO) BMDCs were transfected with HSV60 (1 μg/ml), VACV70 (1 μg/ml), HT DNA (1 μg/ml), ISD (1 μg/ml), cGAMP (1 μg/ml) and poly(dA:dT) (1 μg/ml) separately for 8 h. The cells were lysed for real-time PCR analysis. (F) Wild-type (WT) and RNF90-deficient (KO) BMDCs were infected with HSV-1 for 24 h. The supernatants were collected and subjected to ELISA analysis. (G) Wild-type (WT) and RNF90-deficient (KO) BMDCs were transfected with HSV60 (1 μg/ml), poly(dA:dT) (1 μg/ml), ISD (1 μg/ml), and HT DNA (1 μg/ml) separately for 24 h. The supernatants were collected and subjected to ELISA analysis. β-actin served as a loading control in all the immunoblot assays. The data are representative of three independent experiments and are presented as mean ± SEM. *p < 0.05, **p < 0.01, ***p < 0.001.

To further demonstrate the role of RNF90 in innate immune response, we generated RNF90-deficient mice, in which the third exon was targeted by CRISPR/Cas9 strategy. We cultured BMDCs from wild-type and RNF90-deficient mice and explored the role of RNF90 deficiency in DNA virus- or cytosolic DNA-induced innate immune responses. As shown in Fig 6A, upon HSV-1 infection, compared to wild-type BMDCs, both the MITA expression and the phosphorylation of TBK1, IRF3, p65 and IκB-α increased in RNF90-deficient BMDCs. Similar results were observed in RNF90-deficient BMDCs upon the stimulation of HSV60 (Fig 6B) or HT DNA (Fig 6C) transfection. Moreover, after HSV-1 infection, compared to wild-type BMDCs, RNF90-deficient BMDCs exhibited lower level of K48-linked ubiquitination of MITA whereas no significant difference was observed in K63-linked ubiquitination of MITA (Fig 6D). Additionally, upon the stimulation of cytosolic DNA, such as HSV60, VACV70, HT DNA, ISD, cGAMP and poly(dA:dT), the expression levels of IFN-β, IP-10 and TNF-α were elevated in RNF90-deficient BMDCs (Fig 6E). Similar results were observed in RNF90-deficient BMMs and MEFs (S5A and S6A Figs). Furthermore, the elevation could be inhibited by RNF90 transfection, but not by C29, 32A mutant (S6B Fig). Consistently, ELISA analysis indicated higher expression levels of IFN-β and TNF-α in RNF90-deficient BMDCs and BMMs upon stimulation of HSV-1 or different types of cytosolic DNA (Fig 6F and 6G, S5B and S5C Fig). Additionally, the decrease of HSV-1 titer could be observed in RNF90-deficient BMMs (S5D Fig). Taken together, our findings in RNF90-deficient cells confirmed the inhibitory role of RNF90 in DNA virus- or cytosolic DNA-triggered innate immune responses.

### RNF90 deficiency protects mice from DNA virus infection

**Fig 7:**
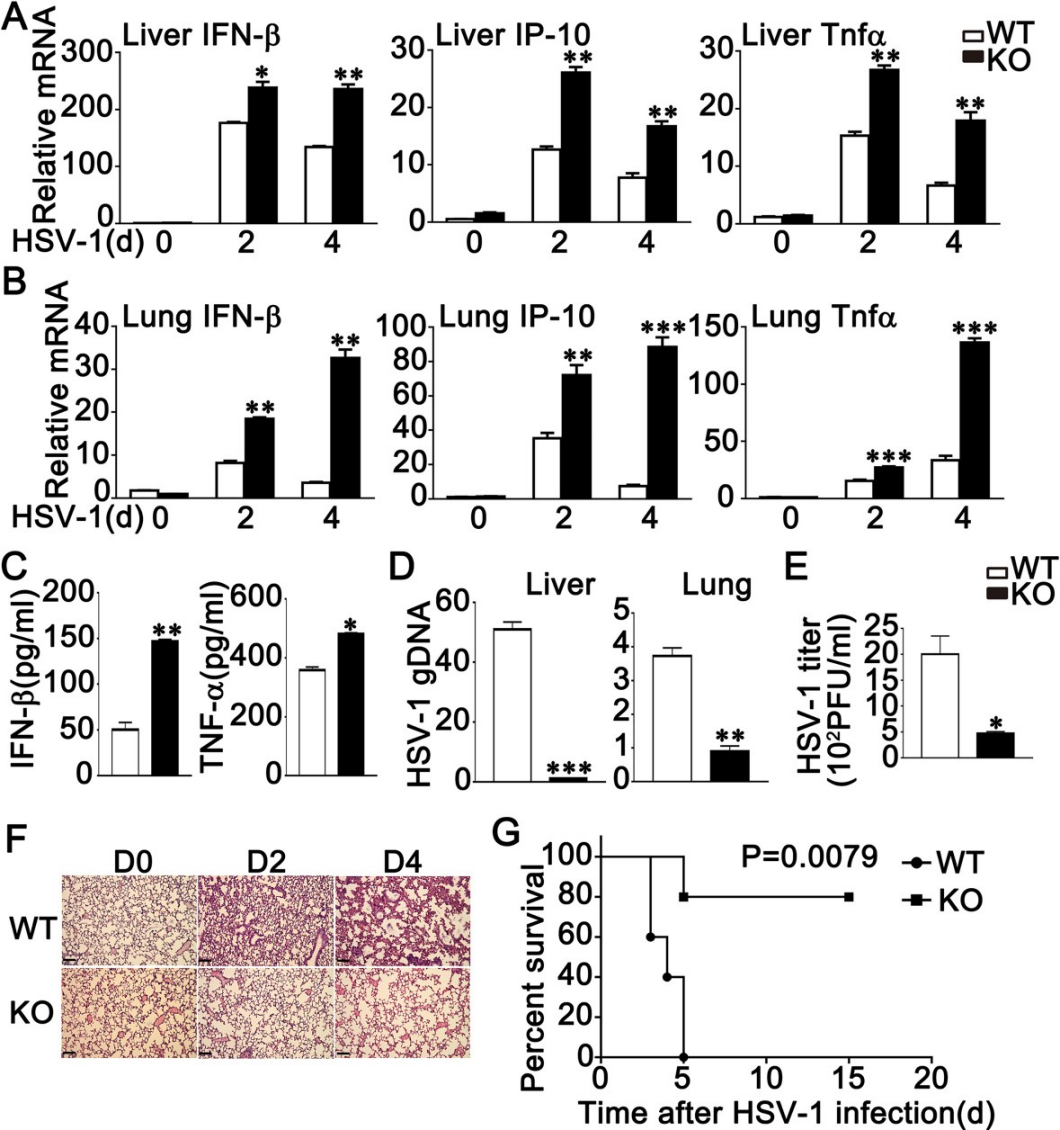
RNF90 deficiency protects mice from DNA virus infection. (A, B) Wild-type (WT) and RNF90-deficient (KO) mice were intravenously infected with HSV-1 (1×10^7^ plaque-forming units (PFU)) for the indicated periods of time, and then the livers (A) and lungs (B) of the mice were subjected to real-time PCR analysis. (C) ELISA of IFN-β and TNF-α in serum of wild-type (WT) and RNF90-deficient (KO) mice 6 h after intravenous infection with HSV-1 (1×10^7^ PFU). (D) Wild-type (WT) and RNF90-deficient (KO) mice were intravenously infected with HSV-1 (1×10^7^ PFU). At 48 h after injection, the HSV-1 genomic DNA in the liver and lung was analyzed by real-time PCR. (E) Wild-type (WT) and RNF90-deficient (KO) mice were intraperitoneally injected with HSV-1 (1×10^7^ PFU). At 20 h after injection, HSV-1 titers in peritoneal wash fluid were measured by plaque assay. (F) Effects of RNF90-deficiency on HSV-1-induced inflammation in the lungs of mice. Sex and age-matched wild-type (WT) and RNF90-deficient (KO) mice (n = 3, 8 weeks old) were intravenously infected with HSV-1 (1×10^7^ PFU) for the indicated periods of time and lung sections were analyzed by H&E staining. Scale bars, 200 μm. (G) Sex and age-matched wild-type (WT) and RNF90-deficient (KO) mice (n = 5, 8 weeks old) were intravenously infected with HSV-1 (2×10^7^ PFU). Survival of these mice was monitored for 15 days. The data are representative of three independent experiments and are presented as mean ± SEM. *p < 0.05, **p < 0.01, ***p < 0.001.

To further elucidate the role of RNF90 in antiviral immune responses in vivo, wild-type and RNF90-deficient mice were intraperitoneally injected with HSV-1. Various organs and serum were collected to evaluate antiviral immune responses that were triggered by HSV-1. Compared to wild-type mice, the expression levels of IFN-β, IP-10 and TNF-α were higher in livers and lungs from RNF90-deficient mice (Fig 7A and 7B). We also observed higher IFN-β and TNF-α expression levels in the serum of RNF90-deficient mice than that of wild-type mice upon HSV-1 infection (Fig 7C). Moreover, in both liver and lung, compared to wild-type mice, genomic DNA copies of HSV-1 were decreased in RNF90-deficient mice (Fig 7D). The decrease of HSV-1 titer could be observed in peritoneal wash fluid in RNF90-deficient mice (Fig 7E). In addition, the lungs from RNF90-deficient mice exhibited less lung destruction than that of the wild-type mice (Fig 7F). Finally, prolonged survival was observed in the RNF90-deficient mice infected with HSV-1 (Fig 7G). Taken together, our findings demonstrated RNF90 deficiency protected mice from DNA virus infection.

## Discussion

In innate immune responses, RNF90 was previously reported to positively regulate LPS-induced production of type I IFN and proinflammatory cytokines in macrophages. However, in this study, our findings demonstrated a new role of RNF90 in antiviral innate immune responses. Upon the stimulation of DNA virus or cytosolic DNA, RNF90, the expression of which was induced significantly, negatively modulated the cellular antiviral signaling. To prove the negative regulatory role of RNF90, we evaluated the effects of RNF90 in cells with RNF90 overexpression, cells with RNF90 knockdown, RNF90-deficient cells and RNF90-deficient mice. Firstly, RNF90 was transfected into HaCaT cells. We found HaCaT cells with RNF90 overexpression exhibited enhanced HSV-1 replication and impaired innate responses against HSV-1 or cytosolic DNA. Secondly, we silenced RNF90 expression in PMA-THP1 cells using siRNA. RNF90-silenced PMA-THP1 cells showed higher production of type I IFN and proinflammatory cytokines, enhanced activation of IRF3 and p65, and impaired HSV-1 replication upon HSV-1 or cytosolic DNA stimulation. Thirdly, we generated RNF90-deficient mice and cultured BMDCs, BMMs and MEFs from wild-type and RNF90-deficient mice. Consistently, RNF90-deficient cells exhibited potentiated phosphorylation of IRF3 and enhanced production of IFN-β, IP-10 and TNF-α after the stimulation of HSV-1 or cytosolic DNA. Finally, RNF90-deficient mice showed higher expression of type I IFN and proinflammatory cytokine in both livers and lungs. Higher IFN-β expression in the serum, lower HSV-1 titer in the peritoneal wash fluid, attenuated lung injury and increased survival rate was also observed in RNF90-deficient mice. All these data strongly characterized the negative regulatory role of RNF90 in DNA virus- or cytosolic DNA-triggered innate immune responses. It is very interesting that RNF90 plays a positive role in LPS-induced signaling pathway whereas has negative effects on DNA virus-triggered innate immune responses. As far as we know, humans and animals are constantly inoculated with various bacterial and viruses, which can mutually interact in the pathogenesis of disease and this interaction may determine the process of disease. This research gave us a hint that the immune system may use RNF90 to reach a delicate balance among viruses, bacterial and their surroundings. Further research about RNF90 in a specific area that contains both bacterial and viruses, such as upper respiratory tract and gut, may help us to understand the role of RNF90 in host defense better.

How to turn off the MITA-mediated signaling remains poorly understood. Prabakaran et al. have reported ubiquitinated MITA is recruited to autophagosomes and its degradation occurs through autophagy in a p62/SQSTM1-dependent manner [33]. Further study by Gonugunta et al. demonstrated MITA vesicles were sorted to Rab7-positive endolysosomes for trafficking-mediated degradation [34]. Meanwhile, RNF5, TRIM29, and TRIM30a target MITA to promote its ubiquitination and degradation in a proteasome-dependent pattern [29, 35–37], whereas some deubiquitinating enzymes, such as USP20, catalyze deubiquitination of MITA and protect it from degradation [38]. Here, our results clarified RNF90 interacted with MITA and promoted its K48-linked polyubiquitination and subsequent proteasome-dependent degradation. Why does the host immune system need so many kinds of molecules to control the degradation of MITA? Further research about the collaboration of these factors and pathways responsible for degradation of MITA is needed to address this question, especially in the process of some specific human diseases.

Except for the effects in antiviral innate immunity, emerging evidences reveal the involvement of MITA in antitumor immune response and MITA agonists are now being extensively developed as a new strategy for cancer treatment [39]. Whether RNF90 is expressed in cancer cells and its effects on MITA-mediated signaling in cancer cells remains unknown. Further clarification of the role of RNF90 in MITA pathway in cancer cells might shed light on the development of new cancer therapeutics.

In summary, our findings indicate that RNF90 is a new negative regulator of MITA-mediated innate immune responses against DNA virus. The expression of RNF90 is induced by viral infection and targets MITA to promotes its K48-linked ubiquitination and proteasome-dependent degradation. These data demonstrated a new molecular involved in the regulation and termination of MITA-mediated antiviral responses.

## Materials and methods

### Ethics statement

C57BL/6 mice were purchased from the Beijing Vital River Laboratory Animal Technology Co., Ltd. All mice were bred and kept in specific pathogen-free (SPF) conditions in the Xinxiang Medical University. All animal care and use protocols were performed in accordance with the Regulations for the Administration of Affairs Concerning Experimental Animals approved by the State Council of People's Republic of China. The animal experiments were approved by the committee on animal care at Xinxiang Medical University (Approval Number: XXMUSPF2017-0045).

### Mice

RNF90-deficient mice were generated on a C57BL/6 background by Laboratory of Genetic Regulators in the Immune System in Xinxiang Medical University through CRISPR/Cas9-mediated gene editing. All animal procedures were performed according to guidelines approved by the committee on animal care at Xinxiang Medical University, China. The age- and sex-matched wild-type and RNF90-deficient mice were used in the experiments.

### cDNA constructs and reagents

Human RNF90 was amplified by PCR using cDNA from HSV-1 stimulated PMA-THP1 cells, and was subsequently cloned into a pcDNA3-Flag/ Myc vector (Invitrogen). All RNF90 deletion mutants were constructed by PCR and subcloned into a pcDNA3 vector. HA-Ubi, HA-K48-Ubi, HA-K63-Ubi, HA-K48R-Ubi, HA-K63R-Ubi, pISRE-Luc, pIFN-β -Luc, HA/Myc-MITA were obtained as described previously [37, 40]. HA-K6-Ubi (22900), HA-K11-Ubi (22901), HA-K27-Ubi (22902), HA-K29-Ubi (22903), HA-K33-Ubi (17607) were purchased form Addgene.

The following antibodies were used for immunoblot analysis or immunoprecipitation: anti-Flag (F3165, Sigma-Aldrich), anti-HA (CO-MMS-101R, Covance), anti-Myc (66004-1-Ig, Proteintech), anti- RNF90 (GTX24541, GeneTex; sc-109107, Santa Cruz; ab170538, Abcam), anti-p-IRF3 (4947, Cell Signaling Technology), anti-IRF3 (sc-9082, Santa Cruz Biotechnology), anti-p-p65 (3033, Cell Signaling Technology), anti-p65 (10745-1-AP, Proteintech), anti-MITA (19851-1-AP, Proteintech), anti-Ubi (sc-8017, Santa Cruz), anti-Ubi-K48 (05–1307, Millipore), anti-Ubi-K63 (05–1313, Millipore), and anti-β-actin (60008–1, Proteintech). The ISD (tlrl-isdn), poly(dA:dT) (tlrl-patn), HSV60 (tlrl-hsv60n), cGAMP (tlrl-nacga23) and poly(I:C) (tlrl-picw) were obtained from InvivoGen. and Herring testis (HT) DNA (D6898) were purchased from Sigma. VACV70 was synthesized by Sangon Biotech. The sequence was as follows: 5’-CCATCAGAAAGAGGTTTAATATTTTTGTGAGACCATGGAAGAGAGAAAGAGATAAAACTTTTTTACGACT-3’. The PMA (S1819) was purchased from Beyotime Biotechnology. MG132 (474790) was obtained from Millipore. 3-MA (M9281) and NH4Cl (A9434) were obtained from Sigma-Aldrich.

### Cell culture, transfection and stimulation

Human Embryonic Kidney (HEK) 293, HEK293T and Tohoku Hospital Pediatrics (THP) 1 cells were kindly provided by Stem Cell Bank, Chinese Academy of Sciences. HaCaT keratinocytes were purchased from Procell Life Science & Technology Co., Ltd., (Wuhan, China). HaCaT, HEK293 and HEK293T cells were cultured in Dulbecco’s modified Eagle’s medium (DMEM). THP1 cells were grown in RPMI 1640. PMA-THP1 cells referred to THP1 cells that were pretreated with 100ng/ml PMA for 24 h. All cells were supplemented with 10% FBS (Gibco), 4 mM L-glutamine, 100U/ml penicillin, and 100U/ml streptomycin under humidified conditions with 5% CO_2_ at 37°C. Transfection of HaCaT, HEK293, HEK293T and THP1 cells was performed with Lipofectamine 2000 (Invitrogen) according to the manufacturer’s instructions. The procedure for generating BMDCs has been described previously [40].

### Immunoprecipitation and immunoblot analysis

Immunoprecipitation and immunoblot analysis were performed as described previously [40]. In short, after the treatment, the cells were lysed in lysis buffer containing 1.0% (vol/vol) Nonidet P40, 20 mM Tris-HCl, pH 8.0, 10%(vol/vol) glycerol, 150 mM NaCl, 0.2 mM Na3VO4, 1mM NaF, 0.1 mM sodium pyrophosphate and a protease inhibitor ‘cocktail’ (Roche). After centrifugation for 20 min at 14,000g, supernatants were collected and incubated with the indicated antibodies together with protein A/G Plus-agarose immunoprecipitation reagent (sc-2003, Santa Cruz Biotechnology) at 4°C for 3 h or overnight. After three washes, the immunoprecipitates were boiled in SDS sample buffer for 10 min and analyzed by immunoblot.

### Real-time PCR

Total RNA was extracted from the cultured cells with TRIzol reagent (Invitrogen) as described by the manufacturer. All gene transcripts were quantified by real-time PCR with SYBR Green qPCR Master Mix using a 7500 Fast real-time PCR system (Applied Biosystems). The relative fold induction was calculated using the 2^-△△^Ct method. The primers used for real-time PCR were as follows:

Human IFN-β,

Forward, 5’- CACGACAGCTCTTTCCATGA -3’;

Reverse, 5’- AGCCAGTGCTCGATGAATCT -3’

Human IP-10,

Forward, 5’- GGTGAGAAGAGATGTCTGAATCC -3’;

Reverse, 5’- GTCCATCCTTGGAAGCACTGCA -3’

Human TNF-α,

Forward, 5’- GGCGTGGAGCTGAGAGATAAC -3’;

Reverse, 5’- GGTGTGGGTGAGGAGCACAT -3’

Human RANTES,

Forward, 5’- TACACCAGTGGCAAGTGCTC -3’;

Reverse, 5’- ACACACTTGGCGGTTCTTTC -3’

Human ISG56,

Forward, 5’- GCCATTTTCTTTGCTTCCCCTA -3’;

Reverse, 5’- TGCCCTTTTGTAGCCTCCTTG -3’

Human RNF90,

Forward, 5’- GCAGCATGGCGAACCCTT-3’;

Reverse, 5’- TGGACCGGAACACCTCACAG -3’

Human MITA,

Forward, 5’- CCAGAGCACACTCTCCGGTA-3’;

Reverse, 5’- CGCATTTGGGAGGGAGTAGTA -3’

Human GAPDH,

Forward, 5’-TCAACGACCACTTTGTCAAGCTCA-3’;

Reverse, 5’-GCTGGTGGTCCAGGTCTTACT-3’

Mouse IFN-β,

Forward, 5’- TCCTGCTGTGCTTCTCCACCACA -3’;

Reverse, 5’- AAGTCCGCCCTGTAGGTGAGGTT -3’

Mouse IP-10,

Forward, 5’- ATCATCCCTGCGAGCCTATCCT -3’;

Reverse, 5’- GACCTTTTTTGGCTAAACGCTTTC -3’

Mouse TNF-α,

Forward, 5’- CGTAGGCGATTACAGTCACGG -3’;

Reverse, 5’- GACCAGGCTGTCGCTACATCA -3’

Mouse GAPDH,

Forward, 5’- ACGGCCGCATCTTCTTGTGCA-3’;

Reverse, 5’- ACGGCCAAATCCGTTCACACC-3’.

### ELISA

BMDCs were infected with viruses or transfected with synthetic nucleic acids for 24 h. The culture media were collected for measurement of IFN-β (PBL) and TNF-α (Thermo Fisher Scientific). 8-week-old wildtype and RNF90-deficient mice were intravenous infected with HSV-1 for 6 h, and then the serum of mice was collected for measurement of IFN-β and TNF-α by ELISA.

### RNA interference

RNF90 Stealth-RNAi siRNA was designed by the Invitrogen BLOCKiT RNAi Designer. The small interfering RNA (siRNA) sequences used were as follows:

R1,

Forward, 5’-GCCUCAUCCUCUCUCUGGAUCUUAA-3’;

Reverse, 5’-UUAAGAUCCAGAGAGAGGAUGAGGC-3’

R2,

Forward, 5’-GAGGACUGUGAGGUGUUCCGGUCCA-3’;

Reverse, 5’-UGGACCGGAACACCUCACAGUCCUC -3’

R3,

Forward, 5’-CAGUCUCUUCUGAGAUGAAGAAUAA-3’;

Reverse, 5’-UUAUUCUUCAUCUCAGAAGAGACUG -3’

The Silencer Select negative control siRNA was purchased from Invitrogen (Catalog no.4390843). PMA-THP1, or HaCaT cells were transfected with siRNA using Lipofectamine 2000 according to the manufacturer’s instructions. At 24 h after transfection, the cells were used for further experiments.

### Viruses and infection

Cells were infected with HSV-1 (KOS strain, multiplicity of infection of 10) for 1.5 h. Then the cells were washed with PBS and cultured in fresh media. For the in vivo study, age- and sex-matched groups of mice were intravenously or intraperitoneally infected with HSV-1. HSV-1 viral titer was determined by the plaque-forming assay on Vero cells.

### In vitro ubiquitination assay

MITA, RNF90 and its mutants were expressed with a TNT Quick-coupled Transcription/ Translation Systems kit (L1171, Promega). In vitro ubiquitination assay was performed with a ubiquitination kit (BML-UW9920, Enzo Life Science) following the manufacturer’s instructions.

### Luciferase reporter gene assay

Luciferase reporter gene assays were performed as described previously[41]. Briefly, HEK293 cells were transfected with an IFN-β/ISRE luciferase reporter plasmid and a Renilla luciferase plasmid as an internal control plus the indicated plasmids. Empty control vector was added so that a total of 1 μg of DNA was transfected into each well of cells. 24 h after transfection, cells were lysed, and reporter activity was analyzed with the Dual-Luciferase Reporter Assay system (Promega).

### Confocal microscopy

After treatment, HaCaT cells were fixed with 4% PFA in PBS, permeabilized with Triton X-100, and then blocked with 1% BSA in PBS. Nuclei were stained with 4, 6-diamidino-2-phenylindole (DAPI).

### Statistics

The data are presented as the means±SD from at least three independent experiments. The statistical comparisons between the different treatments were performed using the unpaired Student t test, and *p*<0.05 was considered statistically significant.

## Supporting information

S1 Fig Effects of RNF90 on HSV-1- or cGAMP-induced innate immune responses. (A) Luciferase activity in HaCaT cells transfected with IFN-β, ISRE or NF-κB luciferase reporter, together with the increasing amounts of RNF90 plasmid as indicated, and then infected with HSV-1 or left untreated (Mock) for 24 h. (B) HaCaT cells were transfected with the empty vector (Vec) or the RNF90 plasmid and then stimulated with cGAMP for 8 h. The cells were lysed for real-time PCR analysis. (C) PMA-THP1 cells were transfected with control siRNA (SC) or RNF90-specific siRNA (R1, R2 and R3) for 24 h, and then stimulated with cGAMP for 8 h. The cells were lysed for real-time PCR analysis. The data are representative of three independent experiments and are presented as mean ± SEM. *p < 0.*05*, **p < 0.*01*.
(TIF)

S2 Fig RNF90 knockdown promotes exogenous cytosolic DNA- induced innate immune responses. (A, B) HaCaT cells were transfected with control siRNA (SC) or RNF90-specific siRNA (R3) for 24 h, and then stimulated with HSV-1 (A) or HSV-60 (B) for 4 h. The cells were lysed for immunoblot assays. β-actin served as a loading control in all the immunoblot assays. The data are representative of three independent experiments.
(TIF)

S3 Fig Effects of RNF90 on signaling mediated by cGAS, TBK1, IRF3. (A) HEK293 cells were transfected with the plasmids as indicated. At 24 h after transfection, the cells were lysed for luciferase assay. (B) HEK293 cells were transfected with control siRNA (SC) or RNF90-specific siRNA (R3), together with the plasmids as indicated. At 24 h after transfection, the cells were lysed for luciferase assay. The data are representative of three independent experiments and are presented as mean ± SEM. *p < 0.*05*, **p < 0.*01*.
(TIF)

S4 Fig RNF90 promotes the ubiquitination of MITA. (A) PMA-THP1 cells were transfected with control siRNA (SC) or RNF90-specific siRNA (R3) and then infected with HSV-1 for indicated periods of time. Then the cells were lysed for real-time PCR analyses. (B, C) HEK293T cells were transfected with the indicated plasmids. At 24 h after transfection, the cells were lysed and subjected to immunoprecipitation (IP) and immunoblot (IB) analysis. (D) HEK293T cells were transfected with various combinations of plasmids as indicated. 24 h later, immunoprecipitation (IP) and immunoblot (IB) analysis were performed. (E) Luciferase activity in HEK293 cells transfected with an ISRE luciferase reporter and empty vector or RNF90, together with wild-type and mutant MITA plasmids as indicated. The data are representative of three independent experiments and are presented as mean ± SEM. *p < 0.*05*, **p < 0.*01*.
(TIF)

S5 Fig RNF90 deficiency promotes exogenous cytosolic DNA-triggered innate immune responses in BMMs. (A) Wild-type (WT) and RNF90-deficient (KO) BMMs were stimulated with HSV60 (1 μg/ml), ISD (1 μg/ml), cGAMP (1 μg/ml) and poly(dA:dT) (1 μg/ml) separately for 8 h. The cells were lysed for real-time PCR analysis. (B) Wild-type (WT) and RNF90-deficient (KO) BMMs were transfected with HSV60 (1 μg/ml), poly(dA:dT) (1 μg/ml), ISD (1 μg/ml), and HT DNA (1 μg/ml) separately for 24 h. The supernatants were collected and subjected to ELISA analysis. (C) Wild-type (WT) and RNF90-deficient (KO) BMMs were infected with HSV-1 for 24 h. The supernatants were collected and subjected to ELISA analysis. (D) Wild-type (WT) and RNF90-deficient (KO) BMMs were infected with HSV-1 for 24 h. The titers of HSV-1 were determined by standard plaque assay. The data are representative of three independent experiments and are presented as mean ± SEM. *p < 0.*05*, **p < 0.*01*, ***p < 0.*001*.
(TIF)

S6 Fig RNF90 deficiency promotes exogenous cytosolic DNA-triggered innate immune responses in MEFs. (A) Wild-type (WT) and RNF90-deficient (KO) MEFs were stimulated with HT DNA (1 μg/ml), ISD (1 μg/ml), VACV70 (1 μg/ml) and cGAMP (1 μg/ml) separately for 8 h. The cells were lysed for real-time PCR analysis. (B) RNF90-deficient (KO) MEFs were transfected with the indicated plasmids and then transfected with HT DNA (1 μg/ml), HSV60 (1 μg/ml) and ISD (1 μg/ml) separately for 8 h. The cells were lysed for real-time PCR analysis. The data are representative of three independent experiments and are presented as mean ± SEM. *p < 0.*05*, **p < 0.*01*, ***p < 0.*001*.
(TIF)
