# Supplementary figures and images for: RNF90 negatively regulates cellular antiviral responses by targeting MITA for degradation
Source: PLoS Pathog. 2020 Mar 3;16(3):e1008387. doi: 10.1371/journal.ppat.1008387 (PMC7069649; doi:10.1371/journal.ppat.1008387)

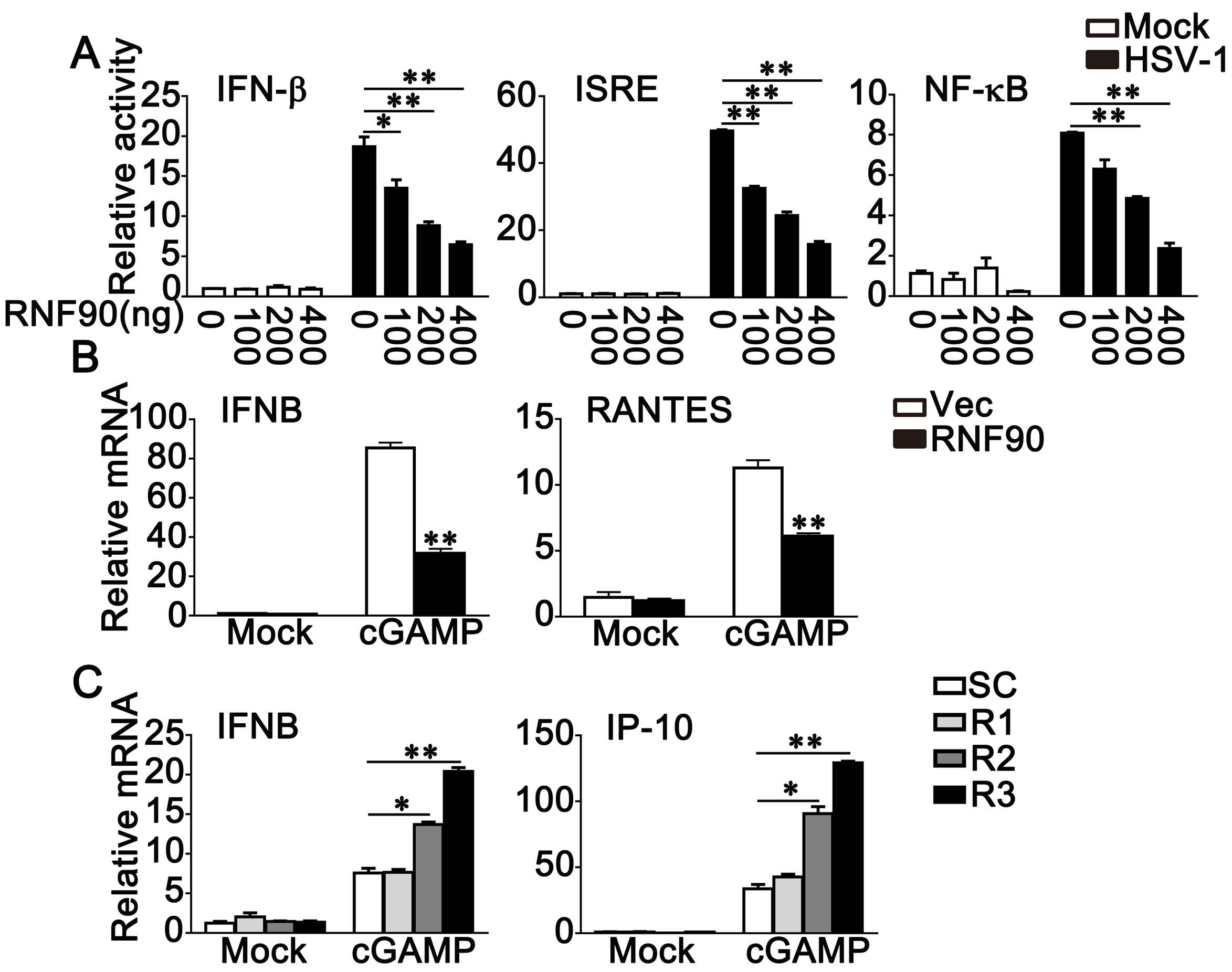

Supplement: S1 Fig — (A) Luciferase activity in HaCaT cells transfected with IFN-β, ISRE or NF-κB luciferase reporter, together with the increasing amounts of RNF90 plasmid as indicated, and then infected with HSV-1 or left untreated (Mock) for 24 h. (B) HaCaT cells were transfected with the empty vector (Vec) or the RNF90 plasmid and then stimulated with cGAMP for 8 h. The cells were lysed for real-time PCR analysis. (C) PMA-THP1 cells were transfected with control siRNA (SC) or RNF90-specific siRNA (R1, R2 and R3) for 24 h, and then stimulated with cGAMP for 8 h. The cells were lysed for real-time PCR analysis. The data are representative of three independent experiments and are presented as mean ± SEM. *p < 0.05, **p < 0.01. (TIF) [file ppat.1008387.s001.tif]

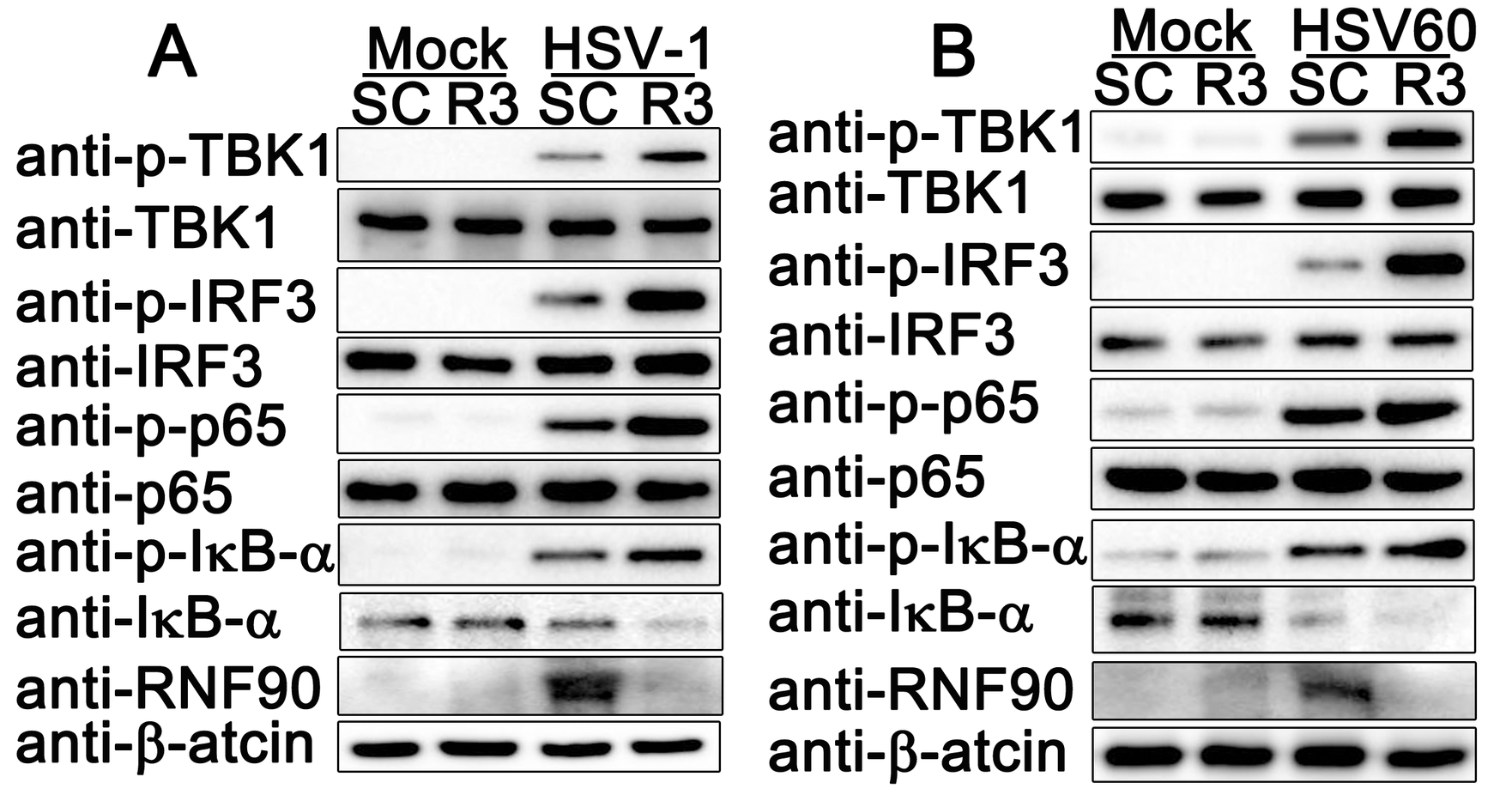

Supplement: S2 Fig — (A, B) HaCaT cells were transfected with control siRNA (SC) or RNF90-specific siRNA (R3) for 24 h, and then stimulated with HSV-1 (A) or HSV-60 (B) for 4 h. The cells were lysed for immunoblot assays. β-actin served as a loading control in all the immunoblot assays. The data are representative of three independent experiments. (TIF) [file ppat.1008387.s002.tif]

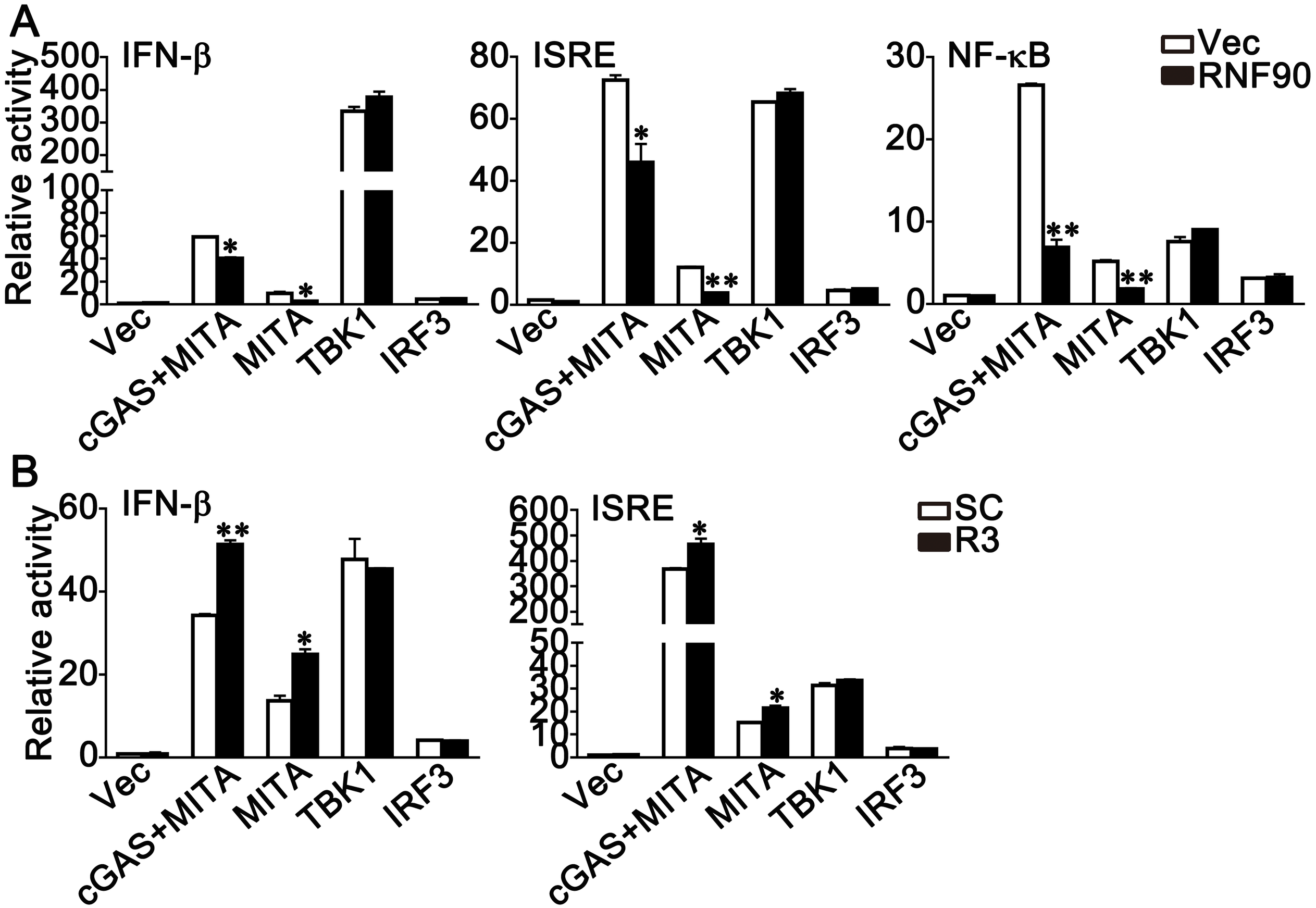

Supplement: S3 Fig — (A) HEK293 cells were transfected with the plasmids as indicated. At 24 h after transfection, the cells were lysed for luciferase assay. (B) HEK293 cells were transfected with control siRNA (SC) or RNF90-specific siRNA (R3), together with the plasmids as indicated. At 24 h after transfection, the cells were lysed for luciferase assay. The data are representative of three independent experiments and are presented as mean ± SEM. *p < 0.05, **p < 0.01. (TIF) [file ppat.1008387.s003.tif]

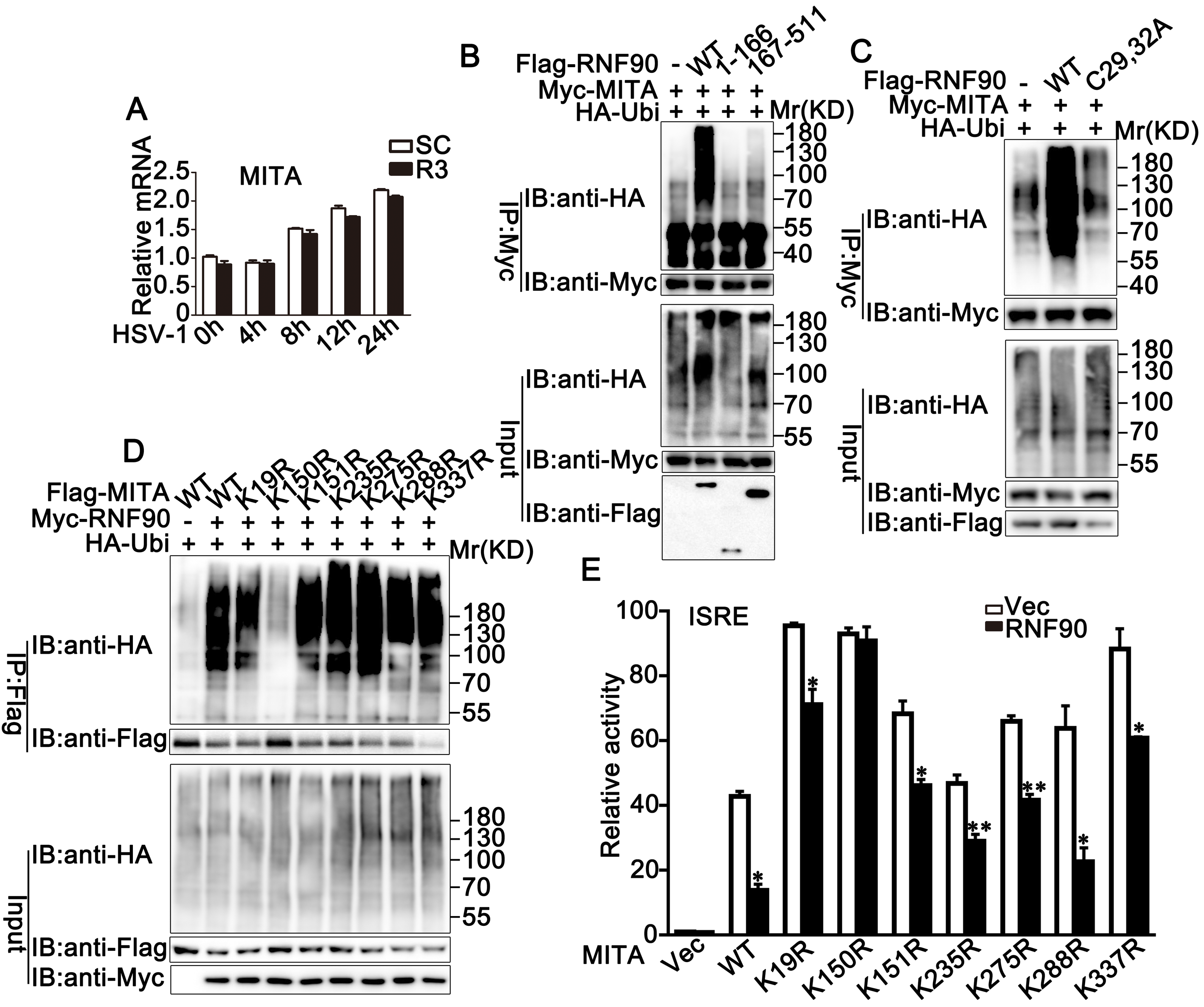

Supplement: S4 Fig — (A) PMA-THP1 cells were transfected with control siRNA (SC) or RNF90-specific siRNA (R3) and then infected with HSV-1 for indicated periods of time. Then the cells were lysed for real-time PCR analyses. (B, C) HEK293T cells were transfected with the indicated plasmids. At 24 h after transfection, the cells were lysed and subjected to immunoprecipitation (IP) and immunoblot (IB) analysis. (D) HEK293T cells were transfected with various combinations of plasmids as indicated. 24 h later, immunoprecipitation (IP) and immunoblot (IB) analysis were performed. (E) Luciferase activity in HEK293 cells transfected with an ISRE luciferase reporter and empty vector or RNF90, together with wild-type and mutant MITA plasmids as indicated. The data are representative of three independent experiments and are presented as mean ± SEM. *p < 0.05, **p < 0.01. (TIF) [file ppat.1008387.s004.tif]

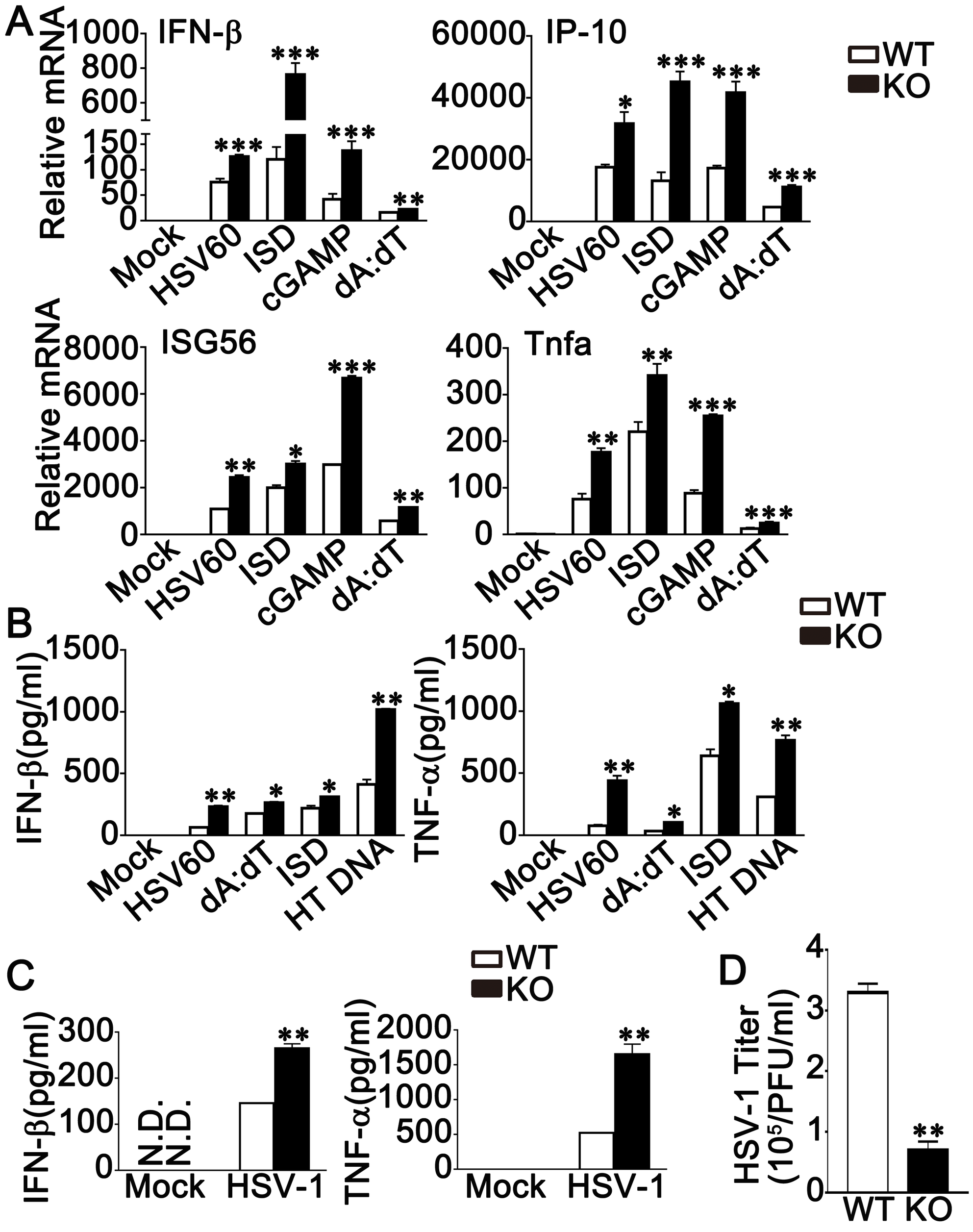

Supplement: S5 Fig — (A) Wild-type (WT) and RNF90-deficient (KO) BMMs were stimulated with HSV60 (1 μg/ml), ISD (1 μg/ml), cGAMP (1 μg/ml) and poly(dA:dT) (1 μg/ml) separately for 8 h. The cells were lysed for real-time PCR analysis. (B) Wild-type (WT) and RNF90-deficient (KO) BMMs were transfected with HSV60 (1 μg/ml), poly(dA:dT) (1 μg/ml), ISD (1 μg/ml), and HT DNA (1 μg/ml) separately for 24 h. The supernatants were collected and subjected to ELISA analysis. (C) Wild-type (WT) and RNF90-deficient (KO) BMMs were infected with HSV-1 for 24 h. The supernatants were collected and subjected to ELISA analysis. (D) Wild-type (WT) and RNF90-deficient (KO) BMMs were infected with HSV-1 for 24 h. The titers of HSV-1 were determined by standard plaque assay. The data are representative of three independent experiments and are presented as mean ± SEM. *p < 0.05, **p < 0.01, ***p < 0.001. (TIF) [file ppat.1008387.s005.tif]

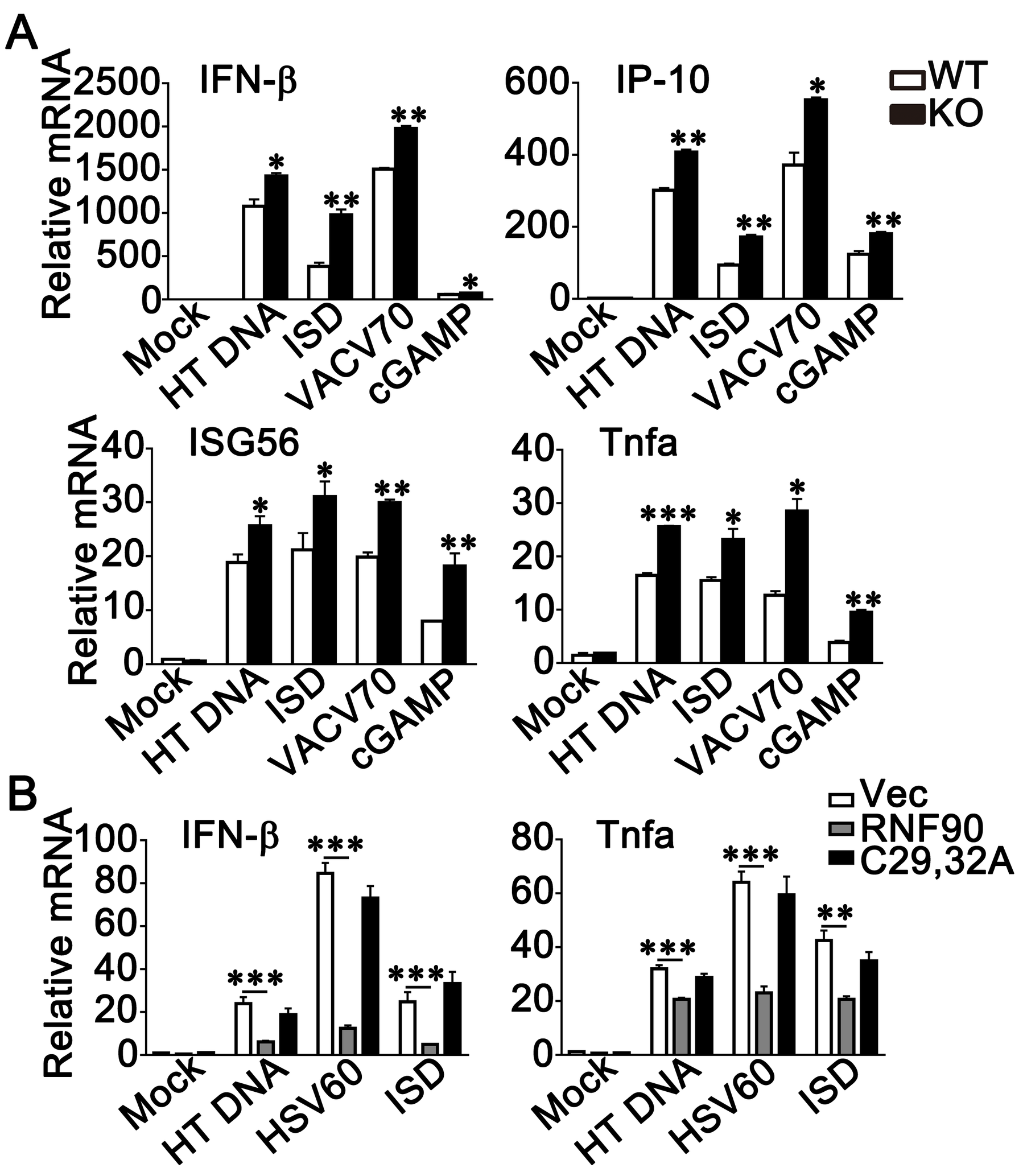

Supplement: S6 Fig — (A) Wild-type (WT) and RNF90-deficient (KO) MEFs were stimulated with HT DNA (1 μg/ml), ISD (1 μg/ml), VACV70 (1 μg/ml) and cGAMP (1 μg/ml) separately for 8 h. The cells were lysed for real-time PCR analysis. (B) RNF90-deficient (KO) MEFs were transfected with the indicated plasmids and then transfected with HT DNA (1 μg/ml), HSV60 (1 μg/ml) and ISD (1 μg/ml) separately for 8 h. The cells were lysed for real-time PCR analysis. The data are representative of three independent experiments and are presented as mean ± SEM. *p < 0.05, **p < 0.01, ***p < 0.001. (TIF) [file ppat.1008387.s006.tif]
